# Summary of Evidence on Nutritional Management for Patients Undergoing Chemotherapy

**DOI:** 10.1002/cam4.70519

**Published:** 2024-12-19

**Authors:** Xin Dan, Ya‐Lin He, Ya‐Lin Tian, Tang‐Lin Chen, Jia‐Yi Yu

**Affiliations:** ^1^ Department of Radiation Therapy and Chemotherapy for Cancer Nursing, West China Second University Hospital Sichuan University Chengdu Sichuan China; ^2^ Key Laboratory of Birth Defects and Related Diseases of Women and Children (Sichuan University), Ministry of Education Chengdu Sichuan China

**Keywords:** cancer, chemotherapy, evidence summary, management, nutrients

## Abstract

**Objective:**

This paper aims to consolidate the most robust evidence on nutritional strategies for patients undergoing chemotherapy, offering evidence‐based guidance for clinical practice. The review highlights critical evidence gaps in nutritional therapy for advanced gastric cancer (AGC) patients undergoing systemic therapy, integrating findings from both prospective and retrospective studies.

**Method:**

According to the “6S” evidence resource pyramid model, clinical decision‐making tools, guidelines, expert consensus, and systematic reviews on nutritional management for chemotherapy patients were systematically retrieved from national and international databases. The methodological quality of the selected literature was evaluated using AGREE II for guidelines, the JBI Evidence‐Based Healthcare Center's standards for systematic reviews, and expert consensus developed by evidence‐based practice experts.

**Results:**

A total of 47 articles were analyzed, consisting of 12 guidelines, 12 expert consensus statements, and 23 systematic reviews. The findings were categorized into five dimensions: interdisciplinary collaboration, nutritional screening and assessment, nutritional requirements, nutritional therapy, and discharge and follow‐up, resulting in the identification of 62 pieces of relevant evidence.

**Conclusions:**

The study provides comprehensive, evidence‐based recommendations for nutritional management in chemotherapy patients. Application of the evidence should be adapted to specific clinical scenarios, patient conditions, preferences, and expert judgment to ensure both feasibility and relevance in clinical practice.

**Contributions:**

This review consolidates diverse nutritional management strategies into a unified framework, addressing evidence gaps in AGC under systemic therapy. Integrating prospective and retrospective studies with interdisciplinary insights provides evidence‐based recommendations to enhance patient care through personalized and standardized approaches.

## 1 Introduction

Cancer is a growing global public health concern. According to GLOBOCAN 2020, there were 19,292,789 new cancer cases and 9,958,133 cancer‐related deaths worldwide in 2020 [1]. The International Agency for Research on Cancer (IARC) reported that China accounted for approximately 23.7% of the global new cancer cases in 2020, with projections suggesting an increase to 6.5 million new cases by 2040 [2]. This rising cancer burden presents significant challenges for healthcare systems globally [1]. Malnutrition is a common concern among cancer patients, contributing to an estimated 10%–20% of cancer‐related deaths, often independent of tumor progression [3]. Chemotherapy, a key cancer treatment, often causes nutrition‐related side effects, particularly gastrointestinal issues such as nausea, vomiting, diarrhea, oral mucositis, altered taste, gastrointestinal mucosal damage, reduced appetite, and anorexia [4, 5]. These side effects can hinder nutrient intake and absorption, leading to weight loss, muscle and fat depletion, and impaired organ function [6].

Cancer patients undergoing chemotherapy are at increased risk of malnutrition, which can reduce their tolerance to treatment and negatively affect its effectiveness and their survival rates [6, 7]. Nutritional support has become essential to multidisciplinary cancer care, playing a crucial role in enhancing prognosis and quality of life (QoL) [8]. However, cancer‐related malnutrition remains significantly under‐recognized and inadequately addressed in clinical practice worldwide [3]. In recent years, major global nutrition associations have issued several authoritative guidelines and expert consensus statements [3, 6, 9, 10, 11, 12]. However, systematic nutritional management strategies specifically for chemotherapy patients are lacking in the current evidence, limiting healthcare providers' ability to access comprehensive and timely information. Matsui et al. [7] reported that malnutrition defined by the GLIM criteria significantly affects adjuvant chemotherapy compliance and relapse‐free survival in advanced gastric cancer (AGC) patients. These findings highlight the urgent need for structured nutritional support during systemic therapy. This study aims to systematically review national and international evidence on the nutritional management of chemotherapy patients, providing insights to enhance their nutritional care during treatment.

## 2 Materials and Methods

### 2.1 Identify Research Questions

Using the PIPOST tool developed by the Joanna Briggs Institute (JBI) Collaborating Centre for Evidence‐based Nursing, this study formulates the evidence‐based research question as follows: The population (P) includes cancer chemotherapy patients (≥ 18 years). The intervention (I) focuses on strategies to improve the nutritional status of chemotherapy patients. Practitioners (P) encompass doctors, nurses, and nutritionists. Outcomes (O) at the patient level include nutrition‐related indicators, complication rates, and length of hospital stay (LOS). At the practitioner level, outcomes include awareness, acceptance, and implementation of evidence‐based practices, while at the system level, outcomes pertain to the adoption of new processes and standards for nutritional management. The settings (S) cover oncology wards, outpatient clinics, communities, and home care environments. The types of evidence (T) considered include clinical decision‐making tools, guidelines, evidence summaries, systematic reviews, and expert consensus.

### 2.2 Retrieval Strategies

In line with the “6S” evidence‐based medicine pyramid model, the search was conducted beginning at the highest level of evidence and proceeding downward. Databases searched included UpToDate, BMJ Best Practice, National Guideline Clearinghouse (NGC), Guidelines International Network (GIN), National Institute for Health and Care Excellence (NICE), American Society for Parenteral and Enteral Nutrition (ASPEN), European Society for Clinical Nutrition and Metabolism (ESPEN), Chinese Society for Parenteral and Enteral Nutrition (CSPEN), Cochrane Library, PubMed, Web of Science, Embase, China Biology Medicine disc (CBMdisc), Wanfang Database, and China National Knowledge Infrastructure (CNKI). Search terms are constructed using a combination of MeSH terms and free‐text keywords, including: “Neoplasms,” “Tumor,” “Oncology,” “Cancer,” “Pharmacologic therapy,” “Chemotherapy,” “Drug therapies,” “Malnutrition,” “Nutrition,” “Diet therapy,” “Nutrition management,” “Dietary management,” “Nutrition assessment,” “Enteral nutrition,” “Parenteral nutrition,” “Nasogastric tube,” and “Nutritional Status.” Detailed search strategies for both Chinese and English sources are provided in the Supporting Information.

### 2.3 Criteria for Inclusion and Exclusion

Inclusion criteria: (1) Research subjects are cancer chemotherapy patients, aged ≥ 18 years; (2) Focus on the nutritional management of chemotherapy patients, including nutritional risk screening, assessment, and intervention. Inclusion criteria also encompassed prospective and retrospective studies evaluating the outcomes of nutritional interventions in AGC patients undergoing systemic therapy; (3) Evidence types include clinical decision‐making tools, guidelines, evidence summaries, systematic reviews, and expert consensus; (4) Outcome indicators include nutrition‐related indicators, incidence of complications, length of hospital stay (LOS), etc.; (6) Publications in Chinese or English are accepted; (7) Literature search period is from January 2014 to May 2024. Exclusion criteria: (1) Incomplete information and duplicate publications; (2) Literature without full‐text access; (3) Low‐quality literature, defined as guidelines rated as Grade C, systematic reviews with a methodological quality score ≤ 4 points, or expert consensus with 50% of entries rated “No” or 80% rated “Unclear”; (4) Old versions of guidelines, consensus, etc., when updated versions are available; (5) Traditional reviews, or systematic review protocols.

### 2.4 Literature Quality Evaluation

#### 2.4.1 Guidelines

Four researchers independently evaluated the methodological quality of the included guidelines using the 2017 version of the Appraisal of Guidelines for Research and Evaluation II (AGREE II) [13]. The consistency among evaluators was determined using the Intra‐class Correlation Coefficient (ICC), where an ICC below 0.40 indicates poor consistency, values between 0.45 and 0.75 indicate good consistency, and values above 0.75 indicate high consistency [14]. AGREE II evaluates six dimensions across 23 items, with scores ranging from 1 (strongly disagree) to 7 (strongly agree). Guidelines were included or excluded based on standardized score results. The guideline is strongly recommended if all dimensions have standardized scores of ≥ 60% (Grade A). It is recommended if three or more dimensions score ≥ 60% and fewer than three dimensions score ≤ 30% (Grade B). It is not recommended if three or more dimensions score < 30% (Grade C).

#### 2.4.2 Expert Consensus

The Joanna Briggs Institute (JBI) Critical Appraisal Checklist for Text and Opinion Papers (2016 edition) was used to evaluate the quality of expert consensus/opinions. This tool evaluates six domains, with each domain scored as “yes” (1 point), “no” (0 points), or “unclear” (0 points).

#### 2.4.3 Systematic Reviews

The methodological quality of the systematic review was assessed using the A Measurement Tool to Assess the Methodological Quality of Systematic Reviews (AMSTAR) [15]. This tool consists of 11 domains, with each domain scored as “yes,” “no,” or “unclear,” with “yes” responses receiving 1 point, while “no” or “unclear” responses score 0 points. Total scores range from 0 to 11, with the overall quality categorized as low (0–4 points), moderate (5–8 points), or high (9–11 points).

### 2.5 Evidence Classification Standards

The JBI Evidence Pre‐ranking and Evidence Recommendation Grade System (2014 edition) was applied to determine the evidence level and recommendation strength [16]. Evidence was ranked from Level I to Level V, depending on the type of study. When multiple sources (e.g., guidelines, expert consensus, and systematic reviews) supported the same finding, the highest evidence level was used, referencing the JBI Pre‐Ranking list for interventional studies. The FAME framework (Feasible, Appropriate, Meaningful, Effective) was applied to determine recommendation strength, categorized as either Level A (strong recommendation) or Level B (weak recommendation). After the initial grading, the GRADE tool was used for systematic reviews to further upgrade or downgrade the quality and strength of evidence. Four researchers independently evaluated the evidence level, and any disagreements were resolved through group discussions involving five senior experts in evidence‐based medicine.

## 3 Results

### 3.1 Literature Retrieval Process and General Characteristics of the Included Literature

**FIGURE 1:**
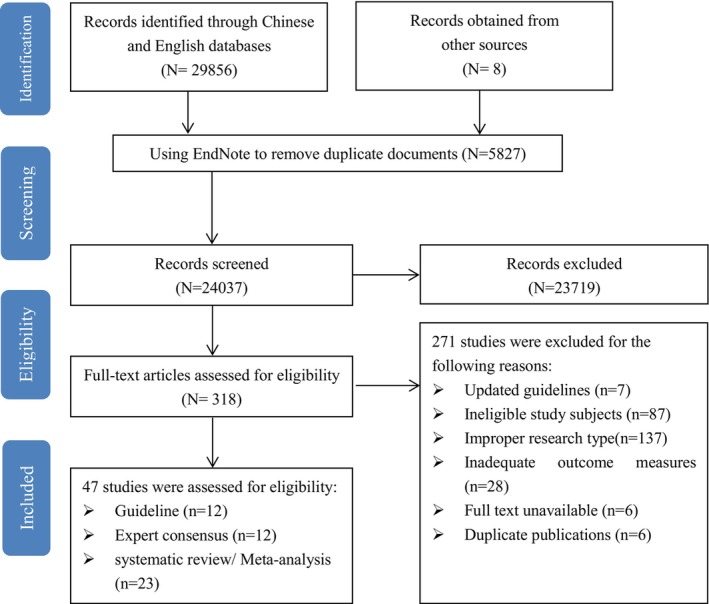
PRISMA flowchart of paper selection.

The process of literature screening is detailed in Figure 1. An initial search yielded 29,856 articles from both Chinese and English databases, with an additional eight articles identified through reference tracking and snowball sampling. After removing 5827 duplicates using EndNote X9 software, 24,037 articles remained for title and abstract screening. During this phase, 23,719 articles were excluded based on criteria such as irrelevant subjects, study types, designs, or outcome measures. The remaining 318 articles underwent full‐text review, and 271 were excluded due to outdated guidelines, non‐compliant subjects or study types, insufficient outcome data, inaccessible full texts, or duplicate publications. Ultimately, 47 articles were included in the analysis, consisting of 12 guidelines, 12 expert consensus documents, and 23 systematic reviews or meta‐analyses. The essential characteristics of the included literature are presented in Supporting Information.

### 3.2 Evaluation of Publication Quality

#### 3.2.1 Quality Evaluation of Guidelines

This study included 12 guidelines, two from ESPEN [3, 17], one each from the ASPEN [9], the American College of Gastroenterology (ACG) [11], the Academy of Nutrition and Dietetics (AND) [12], and the French Speaking Society of Clinical Nutrition and Metabolism (SFNEP) [10], with the remaining six issued by the China Anti‐Cancer Association (CACA) [5, 18, 19, 20, 21] and CSPEN [6]. Quality was assessed using the AGREE II instrument, with scores across six domains as follows: Scope and Purpose (72.22%–94.44%); Stakeholder Involvement (41.67%–92.43%); Rigor of Development (47.40%–97.92%); Clarity of Presentation (63.54%–91.67%); Applicability (56.25%–97.23%); and Editorial Independence (30.83%–95.82%). Six guidelines received a Grade A rating, indicating standardized scores above 60% in all six domains. The remaining six guidelines were rated as Grade B, with standardized scores above 30% in all domains and at least three domains scoring above 60% (Table 1).

**TABLE 1:** Quality evaluation results of guidelines.

Included literature	Percentage of standardization in each domain of the guide (%)						Number of fields ≥ 60%	Number of fields ≥ 30%	Recommendation grade
	Scope and Grade purpose	Stakeholder Involvement	Rigor of Development	Clarity of Presentation	Applicability	Editorial Independence			
Arends (2017) [17]	94.44	92.43	97.92	88.89	97.23	95.82	6	6	A
McClave (2016) [11]	87.50	72.22	79.80	81.94	92.66	88.47	6	6	A
CSPEN (2017) [6]	94.44	87.50	80.21	90.28	81.94	79.19	6	6	A
Muscaritoli (2021) [3]	86.11	72.22	87.50	81.94	93.06	61.11	6	6	A
Thompson (2017) [12]	87.50	86.11	87.50	91.67	90.28	79.19	6	6	A
August (2009) [9]	85.53	72.18	80.21	75.32	76.49	73.25	6	6	A
CACA (2023) [21]	87.50	61.11	47.40	80.21	72.22	63.54	5	6	B
SFNEP (2014) [10]	72.22	67.19	51.39	81.94	72.22	90.28	5	6	B
Cui (2020) [18]	72.22	86.11	67.19	63.54	56.25	46.88	4	6	B
CACA (2016) [5]	86.11	48.61	47.40	67.19	81.94	56.25	3	6	B
CACA (2016) [19]	72.22	41.67	47.40	67.19	63.54	54.17	3	6	B
CACA (2016) [20]	77.78	41.67	51.25	79.17	62.71	30.83	3	6	B

Abbreviations: CACA, China Anti‐Cancer Association; CSPEN, Chinese Society for Parenteral and Enteral Nutrition; SFNEP, French Speaking Society of Clinical Nutrition and Metabolism.

#### 3.2.2 Quality Evaluation of Expert Consensuses

This study analyzed 12 expert consensus papers: one from Spain [22], two from Taiwan [23, 24], and nine from mainland China [25, 26, 27, 28, 29, 30, 31, 32, 33]. Quality was assessed using the JBI Critical Appraisal Checklist for Text and Opinion Papers, with scores ranging from 5 to 6 points. One paper met the criteria in all six domains [22]. Among the remaining 11 papers, two were rated “No” for “whether the sources of viewpoints are clearly indicated” [23, 27], two were rated “Uncertain” for “whether the conclusions are based on the results of analysis,” and five were rated “Uncertain” for “whether existing literature was referenced.” [25, 28, 29, 30, 32] Based on a comprehensive assessment, all papers were deemed acceptable for inclusion (Table 2).

**TABLE 2:** Critical appraisal consensus scores for expert consensus.

Included literature	JBI critical appraisal checklist for text and opinion papers						
	Source of opinion	Standing in field	Population	Analytical process	Extant literature	Incongruence with literature	Critical appraisal score
Carrato (2022) [22]	1	1	1	1	1	1	6
Lin (2018) [24]	1	1	1	1	1	1	6
CSNO (2023) [33]	1	1	1	1	1	1	6
Chen (2018) [23]	0	1	1	1	1	1	5
CSPEN (2017) [25]	1	1	1	1	U	1	5
CSNO (2022) [26]	1	1	1	U	1	1	5
Li (2016) [27]	0	1	1	1	1	1	5
CSNO (2021) [28]	1	1	1	1	U	1	5
CSNO (2021) [29]	1	1	1	1	U	1	5
CSNO (2022) [30]	1	1	1	1	U	1	5
CSNO (2022) [31]	1	1	1	U	1	1	5
CSNO (2023) [32]	1	1	1	1	U	1	5

*Note:* Scoring: 0 – “no,” 1 – “yes,” U – “unclear”.

Abbreviations: CSNO, Chinese Society of Nutritional Oncology; CSPEN, Chinese Society for Parenteral and Enteral Nutrition.

#### 3.2.3 Quality Evaluation of Systematic Reviews

This study included 23 systematic reviews (Table 3) sourced from PubMed (4 studies) [50, 51, 52, 55], Web of Science (9 studies) [34, 35, 42, 43, 46, 47, 53, 54, 56], Embase (5 studies) [36, 37, 44, 48, 49], the Cochrane Library (3 studies) [38, 39, 41], and CNKI (2 studies) [40, 45]. Quality scores ranged from 5 to 11, with 8 studies meeting all AMSTAR tool domains. All studies fulfilled criteria 1, 6, 7, and 8, which involve the review question being clearly stated, critical appraisal, methods to minimize data extraction errors, and methods to combine studies. Except for Kikomeko's [56], all studies provided clear inclusion criteria and search strategies. However, two studies did not conduct comprehensive literature searches [55, 56]. Nine studies were unclear or did not meet the criteria for appraising the quality of included studies [44, 48, 50, 51, 52, 53, 54, 55, 56], and 11 studies were unclear in assessing and reporting the scientific validity of the included studies [43, 46, 47, 49, 50, 51, 52, 53, 54, 55, 56]. One study lacked specific directions for future research [36], and 12 studies showed potential publication bias [42, 46, 47, 48, 49, 50, 51, 52, 53, 54, 55, 56]. Additionally, one study did not provide clear recommendations for policy or practice [45].

**TABLE 3:** Methodological quality of systematic reviews based on AMSTAR scores.

Included literature	JBI systematic reviews and research synthesis											
	Review question stated	Inclusion criteria	Search strategy	Sources/resources	Criteria to appraise	Critical appraisal	Method to minimize errors in data extraction	Methods to combine studies	Publication bias	Recommendations for policy/practice supported	Specific derivative for new research	Criteria appraisal score
Mello (2021) [34]	1	1	1	1	1	1	1	1	1	1	1	11
Miller (2022) [35]	1	1	1	1	1	1	1	1	1	1	1	11
Tao (2022) [36]	1	1	1	1	1	1	1	1	1	1	U	11
Sa‐Nguansai (2024) [37]	1	1	1	1	1	1	1	1	1	1	1	11
Zheng (2020) [38]	1	1	1	1	1	1	1	1	1	1	1	11
Zhang (2021) [39]	1	1	1	1	1	1	1	1	1	1	1	11
Wei (2021) [40]	1	1	1	1	1	1	1	1	1	1	1	11
Wang (2023) [41]	1	1	1	1	1	1	1	1	1	1	1	11
de van der Schueren (2018) [42]	1	1	1	1	1	1	1	1	U	1	1	10
Newell (2021) [43]	1	1	1	1	1	1	1	1	1	1	1	10
Tan (2022) [44]	1	1	1	1	U	1	1	1	1	1	1	10
Chen (2023) [45]	1	1	1	1	1	1	1	1	1	0	1	10
Dambros (2023) [46]	1	1	1	1	1	1	1	1	U	1	1	9
Heilfort (2023) [47]	1	1	1	1	1	1	1	1	U	1	1	9
Sadeghian (2021) [48]	1	1	1	1	U	1	1	1	U	1	1	9
Zeidler (2024) [49]	1	1	1	1	1	1	1	1	U	1	1	9
Benna‐Doyle (2024) [50]	1	1	1	1	U	1	1	1	U	1	1	8
Bossola (2015) [51]	1	1	1	1	U	1	1	1	U	1	1	8
Bossola (2022) [52]	1	1	1	1	U	1	1	1	U	1	1	8
Cintoni (2023) [53]	1	1	1	1	U	1	1	1	U	1	1	8
Limon‐Miro (2017) [54]	1	1	1	1	U	1	1	1	U	1	1	8
Caillet (2017) [55]	1	1	1	0	U	1	1	1	U	1	1	7
Kikomeko (2023) [56]	1	0	0	0	0	1	1	1	U	1	1	5

*Note:* Scoring: 0 – “no,” 1 – “yes,” U – “unclear”.

#### 3.2.4 Summary and Analysis of Best Evidence

Through systematic retrieval and extraction, this study identified 86 pieces of evidence. After team discussions, 14 pieces were consolidated into 3 due to overlap, and 17 were considered redundant, with 4 retained. Ultimately, 62 pieces of evidence related to the nutritional management of cancer chemotherapy patients were compiled. These findings span five major themes: interdisciplinary collaboration, nutritional screening and assessment, nutritional requirements, nutritional therapy, and discharge and follow‐up (Table 4).

**TABLE 4:** Summary of the best evidence on nutritional management for patients undergoing chemotherapy.

Category	Recommendation	Evidence level	Recommendation grade
Interdisciplinary collaboration	1. Nutritionists should collaborate with other healthcare professionals, managers, and public policymakers to ensure that nutritional assessment becomes a crucial component of the care process for adult oncology patients [12]	II	A
	2. Nutritionists should be integrated members of interdisciplinary teams to provide multimodal nutritional therapy for adult oncology patients undergoing chemotherapy [12]	III	B
	3. Clarify the roles and responsibilities within the multidisciplinary team and improve the nutrition referral system and processes [22]	IV	B
Nutritional screening and assessment	**Timing for screening and assessment**		
	4. Cancer patients should undergo nutritional risk screening immediately upon diagnosis, with regular and repeated screenings during the course of treatment and follow‐up [3, 6, 17, 22]	IV	A
	5. Patients at no risk of malnutrition are recommended to undergo nutritional risk screening once a week during their hospital stay [12, 22]	II	A
	6. Patients at risk of malnutrition should undergo further assessment to determine whether treatable nutritional symptoms and metabolic disorders are present [17]	III	A
	**Screening and assessment tools**		
	7. The NRS‐2002 can be used to screen for nutritional risk among hospitalized cancer patients. Additionally, the MST and MUST are widely used for outpatient cancer patients and elderly cancer patients, respectively [6, 11, 12, 25, 31]	II	A
	8. Positive screening individuals are recommended to undergo nutritional assessment, with PG‐SGA recommended as a multidimensional assessment tool [6, 12, 24, 31]	II	A
	**Content of evaluation**		
	9. Since the cancer diagnosis, regularly assess the patient's dietary intake, including changes in food and fluid/beverage consumption. Evaluate the adequacy and appropriateness of nutrient intake or management, as well as the actual daily intake through enteral and parenteral nutrition. Additionally, assess the use of medications and complementary or alternative medicine products and factors affecting food accessibility [12]	II	A
	10. Regularly assess patients' laboratory data, such as blood glucose, white blood cell count, nutritional anemia analysis (including hemoglobin, hematocrit, folate, vitamin B‐12, and iron levels), as well as liver and kidney function analysis. Evaluate inflammatory responses, including C‐reactive protein levels. Conduct gastrointestinal function tests (such as swallowing ability, abdominal imaging, gastric emptying, and transit time) [12]	II	A
	11. Regularly evaluate the patient's nutritional assessment findings, which include muscle mass, subcutaneous fat, pressure ulcers or wounds, signs of malnutrition, changes in functional indicators (such as Karnofsky performance score and grip strength), and localized or generalized fluid retention [12]	IV	A
	12. Regular assessment of patients' psychological factors (such as depression, fatigue) and socio‐economic factors (such as social support) [3, 12, 17]	IV	A
Nutritional requirements	**Energy**		
	13. Patients who are bedridden are advised to consume 20–25 kcal/(kg day), whereas ambulatory patients should consume 25–30 kcal/(kg day) [5, 6, 17, 29]	III	A
	14. It is suggested not to use dietary restrictions on energy intake in patients with or at risk of malnutrition [3, 17]	III	A
	15. Short‐term fasting (STF) combined with chemotherapy may help reduce treatment side effects and improve QoL [48, 56]	III	B
	**Protein**		
	16. The recommended protein intake is 1.5–2.0 g/(kg day). For individuals with impaired kidney function, it should be restricted to no more than 1.0 or 1.2 g/(kg day) [6, 17]	II	A
	**Fat**		
	17. Increase the proportion of fats as an energy source in the diets and nutritional support formulas for cancer patients, thereby enhancing the energy density of these diets [17, 29]	II	A
	**Trace elements**		
	18. Vitamin and mineral intake should be roughly equal to the recommended daily allowance, and high doses of micronutrients should not be used without specific medical guidance or supervision [17]	III	A
Nutritional therapy	**Principle**		
	19. The nutritional status is satisfactory, and there is no nutritional risk for chemotherapy patients. Hence, routine nutritional intervention is unnecessary [5, 19, 32, 33, 53]	II	A
	20. For chemotherapy patients who are malnourished or at high risk of malnutrition, nutritional therapy should be implemented using a stepped approach [26]	I	A
	21. There is no evidence to suggest that nutritional therapy promotes tumor growth, and this concern does not need to be considered in clinical practice [27]	I	A
	**The timing of nutritional therapy**		
	22. Patients who have lost more than 10% of their body weight and have a PG‐SGA score > 4 points [5]	III	A
	23. Chemotherapy severely affects food intake, with an expected duration of more than 1 week, and chemotherapy cannot be discontinued. Even if discontinued, sufficient dietary recovery cannot be achieved for a longer period of time [5, 27]	II	A
	24. When daily energy intake is < 60% of daily energy expenditure for more than 10 days [5]	III	A
	25. Recent weight loss > 5% due to inadequate intake [5]	III	A
	**Approaches to nutritional therapy**		
	*ONS*		
	26. ONS is the preferred nutritional therapy for patients who can swallow and have normal gastrointestinal function [6, 50, 55]	I	A
	27. For patients with low oral intake, it is recommended to provide individualized nutritional education and dietary guidance in conjunction with oral nutritional supplements [25]	III	A
	28. During chemotherapy, ONS rich in high‐protein n‐3 PUFA have a positive effect on improving weight [37, 42]	II	B
	29. Insufficient and inconsistent clinical data prevent recommending omega‐3 PUFA‐enriched ONS for improving CIPN [21, 41, 45]	IV	A
	30. Whey protein‐enriched ONS can improve serum albumin and immunoglobulin levels during tumor chemotherapy, as well as enhance nutritional status scores [21]	III	A
	*EN*		
	31. In patients receiving preoperative chemotherapy or chemoradiotherapy, EN provides the benefits of maintaining body weight, reducing treatment‐related toxicity, and preventing treatment interruption [23]	II	B
	32. When there is a high nutritional risk, the inability to feed orally, or when ONS are unable to meet the body's nutritional needs, it is recommended to initiate EN [6, 19, 20, 31, 32, 33]	III	A
	33. If oral intake is less than two‐thirds of the required amount, or if persistent enteritis continues for more than 7 days after chemotherapy, EN is recommended [10]	II	B
	34. For patients needing EN, the nasogastric tube is the preferred route. For those unable to tolerate a nasogastric tube or at high risk of reflux and aspiration, a nasojejunal tube should be considered [10, 11, 33]	III	A
	35. If the expected feeding duration is > 4 weeks, it is recommended to use a gastric or jejunal feeding tube [6, 11]	III	B
	36. Prophylactic feeding through a nasogastric tube or percutaneous gastrostomy can prevent weight loss, reduce dehydration and hospitalization, and prevent treatment interruptions in patients [51, 52]	III	A
	37. For EN via a nasogastric tube, using a small‐diameter tube is recommended. For EN via a gastrostomy, if the patient has an unresected tumor in the head, neck, or esophagus, it is not recommended to use pull techniques for placement [10, 33]	III	B
	*PN*		
	38. If EN can meet the normal nutritional requirements, routine PN therapy is not recommended [17].	II	A
	39. PN is chosen when patients experience severe mucositis, uncontrollable vomiting, intestinal obstruction, severe malabsorption, persistent diarrhea, or symptomatic intestinal transplant host disease [10, 17, 27, 51]	III	B
	40. In hospitalized patients receiving PN therapy, mild permissive underfeeding (providing 80% of energy requirements while ensuring adequate protein) should be considered for the first 7–10 days. After the initial week, energy delivery should be increased to meet the targeted energy goals [17]	III	B
	41. When using PN, it is recommended to prevent refeeding syndrome if weight loss exceeds 20%, BMI is < 13, or the duration of use exceeds 15 days [10]	III	A
	42. For patients with tumors requiring prolonged PN for several weeks or more, or with significant cachexia, special nutritional formulas are recommended. It is suggested to use high‐fat, low‐carbohydrate formulas with a sugar/fat ratio of 1:1 [5]	II	A
	43. For tumor patients with expected survival exceeding 3 months and stable condition, in the presence of intestinal obstruction or pseudo‐obstruction, under appropriate conditions, home PN may be considered to meet nutritional needs [6, 22]	IV	B
	44. Early discontinuation of PN should be considered once gastrointestinal function has recovered [31]	II	B
	**Types of nutritional supplements**		
	*Amino acid formulation*		
	45. For chemotherapy patients recommended to undergo EN and PN treatment, it is advisable to utilize complex amino acid preparations that contain a wide range of amino acids [5]	II	A
	46. Patients at risk of hepatic encephalopathy are recommended to use amino acid preparations rich in branched‐chain amino acids [5]	II	A
	*Lipid emulsion*		
	47. Medium/long‐chain triglyceride emulsions may be more suitable for tumor patients receiving PN, especially those with concurrent hepatic dysfunction [5]	III	B
	48. The olive oil‐based fat emulsion has a negligible impact on immune function and liver function. Its moderate content of vitamin E can help reduce lipid peroxidation [5]	II	A
	49. A fish oil fat emulsion rich in ω‐3 PUFA helps reduce the risk of cardiovascular diseases, suppress inflammatory responses, balance immune function, and potentially inhibit tumor growth [5, 36]	III	A
	*Immunomodulators*		
	50. An intestinal immune regulation formula (containing glutamine, arginine, nucleotides, and ω‐3 PUFA, etc.) may help reduce the incidence of chemotherapy‐induced mucositis and diarrhea [5, 12, 23, 24, 31, 38, 44]	III	B
	51. Supplementation with glutamine can improve the nutritional status and immune function of chemotherapy patients and also alleviate the neurotoxicity induced by vincristine [10, 23, 31]	II	B
	52. When severe stress conditions, such as serious infections in chemotherapy patients, arise, the application of immune modulation formulas should follow guidelines pertaining to critical illness [5, 35]	II	A
	*Drug formulation*		
	53. Short‐term use of corticosteroids (1–3 weeks) may be considered to stimulate appetite in advanced cancer patients experiencing anorexia. However, it is important to monitor for side effects such as muscle wasting, insulin resistance, and infections [3, 6, 17]	I	B
	54. Consider using progestogens to increase appetite in patients with advanced‐stage anorexia nervosa, but be mindful of potential serious side effects such as thromboembolism [3, 17]	I	B
	55. Currently, there is a lack of consistent clinical data to reliably recommend the use of anabolic steroids for increasing muscle mass [17]	III	B
	56. Currently, there is insufficient consistent clinical data to recommend cannabinoids for improving taste disorders or anorexia in cancer patients [3, 17]	III	B
	57. Currently, there is insufficient consistent clinical data to recommend the use of non‐steroidal anti‐inflammatory drugs for improving weight loss in cancer patients [17]	III	B
	58. For patients experiencing early satiety, after addressing constipation through diagnosis and treatment, it is recommended to consider the use of prokinetic drugs. However, caution should be exercised due to the potential adverse effects of metoclopramide on the central nervous system and domperidone on cardiac rhythm [3, 17]	II	B
Discharge and follow‐up	59. For patients with moderate to severe malnutrition postoperatively undergoing chemotherapy, it is recommended to continue nutritional therapy after discharge and to regularly follow up and monitor their nutritional status [5]	III	B
	60. After discharge, the preferred nutritional treatment is ONS, primarily consisting of whole protein formulas. It is recommended to take these supplements between meals, and the duration can be maintained for 3–6 months or longer [24, 33]	I	A
	61. Maintain moderate aerobic exercise and/or resistance training within tolerable limits to preserve muscle mass and strength [3, 17, 25, 26]	I	A
	62. It is recommended to maintain a BMI between 18.5 and 25 kg/m^2^ [5]	III	B

Abbreviations: BMI, body mass index; CIPN, chemotherapy‐induced peripheral neuropathy; EN, enteral nutrition; MST, Malnutrition Screening Tool; MUST, Malnutrition Universal Screening Tool; NRS‐2002, Nutrition Risk Screening 2002; ONS, oral nutritional supplement; PG‐SGA, Patient‐Generated Subjective Global Assessment; PN, parenteral nutrition; PUFA, polyunsaturated fatty acids; QoL, quality of life.

## 4 Discussion

### 4.1 Interdisciplinary Collaboration

Nutritional support is a multidisciplinary effort requiring collaboration among physicians, nurses, dietitians, clinical pharmacists, and patients. Support from administrators and policymakers is essential for the development and implementation of effective strategies [6]. The ASPEN guidelines recommend forming specialized interdisciplinary nutrition support teams to ensure safe, efficient, cost‐effective nutritional therapies [57]. As nutritional support is increasingly integrated into clinical practice, stronger interdisciplinary collaboration becomes essential for delivering standardized and effective patient care [58]. Key steps include forming nutrition support teams, engaging in multidisciplinary oncology treatment, exploring therapeutic models integrating oncology nutrition with other specialties, developing personalized nutritional plans for chemotherapy patients, and applying evidence‐based clinical nutrition therapies [59]. This study introduces a novel approach by leveraging the “6S” evidence pyramid model to systematically integrate high‐quality evidence from guidelines, systematic reviews, and expert consensus into a unified framework. This approach addresses the fragmented nature of current nutritional management practices and provides a robust evidence base for personalized interventions. This framework bridges the gap between research and practice by aligning interdisciplinary collaboration with standardized recommendations, facilitating more consistent and effective nutritional care for chemotherapy patients. Moreover, using a continuous multimodal management strategy for high‐risk hospitalized patients and providing outpatient nutritional interventions upon discharge can enhance physiological tolerance, treatment efficacy, QoL, survival outcomes, and overall prognosis for cancer patients [60].

### 4.2 Nutritional Screening and Assessment

Nutritional risk is a critical factor influencing clinical outcomes, closely associated with survival rate, mortality, complications, LOS, cost‐effectiveness, and QoL [6, 10, 11, 17]. Conducting nutritional screening and assessment for chemotherapy patients is essential to understanding their nutritional status and providing targeted nutritional therapy to improve clinical outcomes [22, 23, 24]. It is recommended that all chemotherapy patients undergo screening for malnutrition risk [6]. Patients without risk should undergo weekly nutritional screenings during hospitalization, while those at risk should receive a detailed nutritional assessment [6, 18].

Currently, there is no globally recognized standard tool for nutritional risks screening [6]. The ESPEN guidelines endorse the nutrition risk screening (NRS‐2002) as the preferred screening tool due to its simplicity, practicality, and effectiveness in predicting risk for hospitalized patients, making it widely accepted as a basis for rational nutritional support [6, 10, 17]. For cancer patients, tools such as the Malnutrition Screening Tool (MST) and the Malnutrition Universal Screening Tool (MUST) are commonly used [6]. MST assesses appetite loss, recent weight loss, and the degree of weight loss, making it particularly suitable for outpatient cancer patients [61]. MUST evaluate BMI, weight loss over 3–6 months, and the impact of acute illness, proving especially useful for elderly cancer patients in both community and hospital settings [62]. Following a positive nutritional screening, Patient‐Generated Subjective Global Assessment (PG‐SGA) is recommended for nutritional assessment [6, 14, 22, 29]. PG‐SGA combines patient self‐assessment with healthcare professional evaluations, covering weight, dietary intake, symptoms, physical activity, functional status, disease‐nutrition relationships, metabolic demands, and physical examination. Scores range from 0–1 for no malnutrition, 2–3 for suspected malnutrition, 4–8 for moderate malnutrition, and 9 or higher for severe malnutrition [63, 64].

Patients undergoing chemotherapy experience symptoms such as nausea, vomiting, mucositis, constipation, and diarrhea, which can impact their nutrient intake. Therefore, regular assessment of dietary intake is essential [5]. Weight loss is a critical clinical manifestation of cancer and is closely linked to patient outcomes [6, 65]. Unintentional weight loss exceeding 10% within 3–6 months is a key indicator of severe malnutrition [6]. Regular monitoring of blood glucose, white cell count, nutritional anemia, and liver and kidney function is also recommended [12]. Additionally, metabolic disturbances and systemic inflammation are common pathophysiological changes in cancer patients. Laboratory markers such as serum C‐reactive protein, albumin, prealbumin, and retinol‐binding protein are important for assessing nutritional status during chemotherapy [6]. Physical activity is another important factor in nutritional assessment, and the Karnofsky Performance Status is recommended for evaluating physical condition [6, 12]. Grip strength measurement effectively reflects skeletal muscle loss, offering insights into nutritional status, daily functional abilities, disability severity, and survival predictions. Changes in body composition, including muscle mass, subcutaneous fat, and fluid, provide an accurate reflection of nutritional status, as these components fluctuate with malnutrition [6, 12]. Common methods for body composition analysis include bioelectrical impedance analysis, dual‐energy X‐ray absorptiometry (DEXA), CT, MRI, and total body potassium counting. DEXA and CT are gold standards for accurately measuring body composition [6].

#### 4.2.1 Nutritional Requirements

For chemotherapy patients, recommended energy intake depends on their level of mobility. Bedridden patients are typically advised to consume 20–25 kcal/(kg day), while more active patients need 25–30 kcal/(kg day) [5, 17]. Effective nutritional therapy relies on accurately calculating resting energy expenditure (REE) [5]. Research by Cao et al. revealed that 70.6% of cancer patients experience hypermetabolism, 23.7% have normal metabolic rates, and 5.7% are hypometabolic [66]. Langius et al. found no significant difference in REE between untreated head and neck cancer patients and healthy controls [(22.1 ± 3.5) kcal/kg vs. (21.5 ± 3.3) kcal/kg, *p* = 0.42] [67]. Nixon and colleagues analyzed REE in colorectal and non‐small cell lung cancer patients, comparing them to healthy individuals and patients with other conditions, such as anorexia nervosa, benign gastrointestinal diseases, and chronic lung diseases. Their results showed no significant differences in REE between tumor and non‐tumor patients [68]. Khor et al. and Reeves et al. reported no significant REE differences between patients with solid tumors and healthy controls, suggesting that cancer patients may not require additional energy supplementation based on current evidence [69, 70]. However, research on daily energy expenditure during chemotherapy remains limited and has produced inconsistent findings.

Early guidelines recommended a minimum protein intake of 1.0 g kg^−1^ day^−1^ for cancer patients, with a target intake of 1.2–2.0 g kg^−1^ day^−1^ [22, 24]. However, recent research suggests that increasing protein intake to 1.5–2.0 g kg^−1^ day^−1^ may lead to better outcomes [3, 6]. This recommendation is based on the dose–response relationship between exogenous protein intake, protein synthesis, and lean body mass. When adequate energy is provided, higher protein intake can enhance muscle protein synthesis, correct negative nitrogen balance, and support tissue repair [6]. For patients with acute or chronic renal insufficiency, protein intake should be limited to 1.0–1.2 g kg^−1^ day^−1^ [6, 17]. The optimal ratio of fat to carbohydrates for energy supply in cancer patients remains uncertain. However, due to the systemic inflammation and insulin resistance frequently observed in these patients, glucose uptake and utilization are often impaired, making fat a more suitable primary energy source [71]. Research has shown that cancer patients can effectively utilize exogenous fat, regardless of weight loss [72]. Therefore, from a metabolic perspective, increasing the proportion of fat in the energy supply is beneficial, particularly for patients with insulin resistance.

The ESPEN guidelines recommend providing vitamins and minerals at levels that approximate the recommended daily intake and discourage the use of high‐dose micronutrients unless specific deficiencies are present [3]. Approximately 50% of cancer patients utilize complementary or alternative medical products, with many opting for multivitamin supplements [3]. However, recent large randomized controlled trials (RCTs) and Mendelian randomization (MR) studies have shown that vitamin D supplementation does not prevent bone loss, fractures, falls, cancer incidence, hypertension, or cardiovascular risk in the general population. The effect of vitamin D on mortality reduction in older adults or those with vitamin D deficiency or osteoporosis remains controversial [73]. Additionally, RCTs found that combined vitamin E (400 IU/day) and vitamin C (500 mg/day) supplementation had no impact on cancer incidence over 10 years [74], and long‐term selenium supplementation (200 μg) did not reduce prostate cancer incidence [75]. Our findings suggest that short‐term fasting (STF) for 48–140 h before and 5–56 h after chemotherapy is well‐tolerated, safe, and feasible for patients undergoing different chemotherapy regimens, with the potential to reduce associated side effects [56].

#### 4.2.2 Nutritional Therapy

Current evidence suggests that for patients undergoing conventional chemotherapy, whether for gastrointestinal or non‐gastrointestinal tumors, the impact of nutritional therapy on outcomes is limited. As a result, guidelines from ESPEN, ASPEN, and CSPEN do not recommend routine nutritional therapy for all chemotherapy patients [3, 6, 9]. Nutritional therapy should be initiated in chemotherapy patients under the following conditions: (1) existing malnutrition; (2) anticipated daily intake < 60% of estimated energy expenditure and duration > 10 days, or anticipated inability to eat for > 7 days; (3) patients experiencing recent weight loss > 5% due to inadequate nutritional intake [5]. Recent findings suggest that tailored nutritional support in AGC patients improves systemic therapy tolerance, reduces toxicities, and enhances quality of life. For example, studies have shown that high‐protein diets and immune‐enhancing nutrients can significantly improve survival rates and patient‐reported outcomes [76, 77]. Recent findings suggest that tailored nutritional support in AGC patients improves systemic therapy tolerance, reduces toxicities, and enhances quality of life. Building upon these findings, this review consolidates diverse strategies into a unified framework, incorporating prospective and retrospective evidence to provide actionable recommendations. This framework emphasizes the importance of standardized yet personalized approaches to optimize nutritional therapy, bridging critical gaps in evidence and practice for AGC patients. For chemotherapy patients at high risk of malnutrition or already malnourished, a stepwise approach is recommended: start with nutritional education, then move to oral nutritional supplements (ONS), followed by total enteral nutrition (TEN), partial parenteral nutrition (PPN), and total parenteral nutrition (TPN) as needed [26]. If nutritional needs are not met by 60% within 3–5 days, escalation of nutritional intervention should be considered [3].

The Chinese Anti‐Cancer Association developed the 3 + 3 model, consisting of three main meals and three oral nutritional supplements (ONS) daily, with ONS recommended between meals and after dinner. This approach improves patient compliance and helps achieve nutritional goals [21]. During chemotherapy, oral high‐protein n‐3 polyunsaturated fatty acids (PUFAs) have been shown to positively influence body weight, especially in high‐risk groups such as elderly individuals, those with low baseline weight, women, and non‐Asian patients [37, 42]. A meta‐analysis of 31 studies demonstrated that ω‐3‐fortified ONS can effectively alter the weight, muscle mass, and QoL of cancer patients. However, its effectiveness in reducing chemotherapy‐induced peripheral neuropathy (CIPN) remains uncertain [78].

For patients unable to meet their energy and protein goals through diet or ONS, enteral nutrition (EN) via tube feeding should be prioritized [6, 19, 20, 31, 32, 33]. Meta‐analyses have confirmed several advantages of EN over parenteral nutrition (PN), including shorter hospital stays, higher plasma protein levels, and lower infection rates [79, 80]. Most guidelines recommend EN as the preferred artificial feeding method because it preserves intestinal barrier function, supports immune function, and aids glycemic control [5, 6, 9, 17]. EN can be administered through various routes, such as nasogastric, nasoduodenal, or nasojejunal tubes, or gastric or jejunal fistulas. The choice of feeding route should be based on the patient's condition, feeding duration, mental status, and gastrointestinal function [6]. Tube feeding should begin at low flow rates (20–30 mL/h) based on intestinal tolerance. The target rate should be reached within 72 h for patients who tolerate feeding well, while gradual escalation over 7 days is recommended for those with poor gastrointestinal tolerance [6]. PN should be considered for patients with absolute contraindications to EN, such as mechanical intestinal obstruction, refractory peritonitis, intestinal ischemia, and severe vomiting [3, 5, 6]. Moreover, supplemental PN or TPN should be considered if enteral intake provides < 60% of energy and protein requirements for more than 10 days [5, 6]. For patients with long‐standing or severe malnutrition, careful monitoring of fluid status, electrolytes, vitamins, and trace elements is crucial during the initial phase of artificial feeding. A gradual increase in energy levels is necessary to prevent refeeding syndrome [6, 24]. In stable cancer patients with an expected survival exceeding 3 months, home PN may be an option for cases of intestinal obstruction or pseudo‐obstruction under appropriate conditions [6]. Unlike inpatient artificial nutrition, home nutritional support requires close cooperation between patients and caregivers, introducing potential challenges. Therefore, close collaboration between healthcare providers and patients is critical to ensure the safe and effective delivery of home nutritional support.

For cancer patients requiring prolonged PN over several weeks or those with significant cachexia, specialized nutritional formulas with a high fat‐to‐carbohydrate ratio (up to a 1:1 glucose‐to‐fat ratio, with fat contributing 50% of non‐protein calories) are recommended [5, 6]. Patients receiving EN and PN during chemotherapy should ideally receive a compound amino acid preparation with a comprehensive spectrum of amino acids [5]. Amino acid preparations enriched with branched‐chain amino acids are particularly advantageous for cancer patients, as they help reduce muscle wasting, support liver function, balance aromatic amino acids, and alleviate symptoms like anorexia and early satiety. These formulations are especially suitable for patients at risk of hepatic encephalopathy [81]. Medium‐chain triglyceride (MCT) emulsions, which hydrolyze and oxidize quickly, can improve nitrogen balance and liver tolerance while exerting less impact on lung function and reducing immune suppression compared to long‐chain triglycerides. MCT emulsions may be particularly beneficial for cancer patients on PN, especially those with concurrent hepatic dysfunction [5].

Immunonutrients such as arginine, glutamine, nucleotides, omega‐3 polyunsaturated fatty acids, and probiotics are widely used in the nutritional support of chemotherapy patients. These nutrients have been shown to improve nutritional status, support metabolic processes, enhance immune function, and reduce inflammatory responses [18]. The European Society for Medical Oncology recommends probiotic formulas containing Lactobacillus to prevent diarrhea in pelvic cancer chemotherapy patients [82]. Additionally, the Academy of Nutrition and Dietetics advises using immunonutrients such as glutamine and glutathione for both the prevention and management of CIPN in cancer patients [12]. Supplementation with glutamine and arginine has demonstrated potential benefits, including increasing the local concentration of chemotherapeutic agents within tumors, raising glutathione levels in normal tissues, reducing treatment‐related side effects, and possibly improving overall survival [5]. However, the optimal timing for initiating immunomodulatory agents remains uncertain, and their use is generally recommended in combination with other therapies rather than as standalone treatments [5].

Metabolic regulators such as glucocorticoids and progestogens are recommended to improve appetite, manage metabolic disorders, and enhance QoL in patients, particularly those facing significant appetite loss and severe nausea and vomiting post‐chemotherapy [3, 6, 17]. Glucocorticoids should be used with caution and restricted to short‐term applications due to their potential side effects. When prescribing progestogens, it is important to monitor for the risk of thrombosis during treatment [5]. Androgens, which have fewer adverse effects than glucocorticoids, may promote weight gain, though current evidence does not definitively support their efficacy in increasing muscle mass [3, 5]. They are also less potent than glucocorticoids and progestogens in stimulating appetite [17]. At present, there is insufficient evidence to recommend cannabinoids or non‐steroidal anti‐inflammatory drugs for improving taste disorders or promoting weight gain [3, 17]. Prokinetic agents like metoclopramide or domperidone are commonly used to enhance gastric emptying and relieve feelings of fullness for early satiety symptoms. While generally well‐tolerated, metoclopramide may cause side effects, including drowsiness, depression, hallucinations, extrapyramidal symptoms, and potentially irreversible tardive dyskinesia [6]. Domperidone, particularly when administered intravenously, may prolong the Q‐T interval and lead to torsades de pointes ventricular tachycardia, though the risk of these electrocardiographic changes is low with typical oral doses [3, 17].

### 4.3 Discharge and Follow‐Up

Continuing nutritional therapy after discharge is recommended for postoperative chemotherapy patients with moderate to severe malnutrition [5, 23, 24]. ONS, preferably whole protein formulations, should be taken between meals for 3–6 months or longer [6]. Post‐gastrectomy chemotherapy patients may develop iron, folic acid, vitamin B12, vitamin D, and calcium deficiencies, requiring regular monitoring and supplementation as needed [33]. Prophylactic calcium and vitamin D supplementation is recommended, along with calcium‐rich foods like milk, cheese, sardines, and dark green leafy vegetables [24, 33]. Additionally, moderate aerobic exercise and/or resistance training, within tolerable limits, is encouraged for chemotherapy patients [3, 17, 26]. Exercise should begin with 5–10 min daily and gradually increase to 150 min per week, aiming for moderate intensity where the body produces light sweat without causing fatigue [26]. Considering the strong correlation between BMI and survival outcomes, hospital stay, and overall functional status in cancer patients, maintaining a BMI between 18.5 and 25 kg/m^2^ is recommended for chemotherapy patients [23, 24]. The comprehensive framework presented in this study has significant implications for clinical practice. Standardizing nutritional management strategies ensures more equitable and effective care delivery, particularly for vulnerable patient populations such as those with AGC. The interdisciplinary insights integrated into this framework emphasize its adaptability across diverse clinical settings, paving the way for future research to refine and expand its application.

## 5 Conclusions

This study provides a comprehensive summary of the nutritional management of chemotherapy patients through a systematic literature review, rigorous quality assessment, and evidence‐based recommendations, offering valuable support for clinical decision‐making. The study did not extend the evidence into clinical practice or explore practical strategies for its application. Therefore, it is recommended that the evidence be applied selectively and specifically, considering clinical scenarios, patient conditions, preferences, and expert judgment. This approach ensures the scientific and effective integration of evidence into clinical practice and supports the standardization of nutritional management for chemotherapy patients.

## Author Contributions

**Xin Dan:** conceptualization, methodology, data curation, formal analysis, writing – review and editing, project administration. **Ya‐Lin He:** methodology, data curation. **Ya‐Lin Tian:** methodology, data curation. **Tang‐Lin Chen:** conceptualization, methodology, formal analysis, writing – review and editing, supervision. **Jia‐Yi Yu:** conceptualization, data curation, writing – review and editing, supervision.

## Ethics Statement

The authors have nothing to report.

## Consent

The authors have nothing to report.

## Conflicts of Interest

The authors declare no conflicts of interest.

## Supporting information


Data S1

## Data Availability

All relevant data are included in this article. Further inquiries can be directed to the corresponding author.
