# Supplementary material for: Summary of Evidence on Nutritional Management for Patients Undergoing Chemotherapy
Source: Cancer Med. 2024 Dec 19;13(24):e70519. doi: 10.1002/cam4.70519 (PMC11656406; doi:10.1002/cam4.70519)
Supplement: Supplementary file 1 — Data S1 [file CAM4-13-e70519-s001.docx]

Supplementary Material

Supplementary Table 1 PubMed search strategy

| Number | Search terms |
| --- | --- |
| #1 | Neoplasms[Mesh] |
| #2 | ((((Neoplasms[Title/Abstract]) OR (Tumor[Title/Abstract])) OR (Cancer[Title/Abstract])) OR (malignancy[Title/Abstract])) OR (oncology[Title/Abstract])))) |
| #3  #4  #5  #6  #7  #8  #9  #10 | #1 or #2  "Drug Therapy"[Mesh]  (((((((((pharmacologic therapy[Title/Abstract]) OR (pharmacotherapy[Title/Abstract])) OR (chemotherapy[Title/Abstract])) OR (chemo*[Title/Abstract])) OR (pharmaco*[Title/Abstract])) OR (drug[Title/Abstract])) OR (Therapy, Drug[Title/Abstract])) OR (Drug Therapies[Title/Abstract])))  #4 or #5  (((("Nutritional Status"[Mesh]) OR "Nutrition Therapy"[Mesh]) OR "Malnutrition"[Mesh]) OR "Nutrition Disorders"[Mesh])))  (((((((((((((((Undernutrition[Title/Abstract]) OR (Malnourishment*[Title/Abstract])) OR (Nutrition*[Title/Abstract])) OR (diet therapy[Title/Abstract])) OR (nutrition management[Title/Abstract])) OR (dietary management[Title/Abstract])) OR (diet*[Title/Abstract])) OR (Therapy, Nutrition[Title/Abstract])) OR (Medical Nutrition Therapy[Title/Abstract])) OR (nutrition assessment[Title/Abstract])) OR (nutrition screening[Title/Abstract])) OR (enteral nutrition[Title/Abstract])) OR (parenteral nutrition[Title/Abstract])) OR (nasogastric tube[Title/Abstract]))  #7 or #8  (("2014"[Date - Publication] : "2024"[Date - Publication])) |
| #11 | #3 and #6 and #9 and #10 |

Supplementary Table 2 Wanfang database search strategy

| Number | Search terms |
| --- | --- |
| #1 | 主题:(癌 OR 癌症 OR 肿瘤) |
| #2 | 主题:(化疗 OR 化学治疗 OR 化学疗法) |
| #3 | 主题:( 饮食 OR 营养 OR 营养支持 OR 营养管理 OR 营养不良 OR 肠内营养 OR 肠外营养 OR 营养治疗 OR 营养筛查 OR 营养评估 OR 营养制剂 OR 口服营养) |
| #4  #5 | 发表时间:2014-*  #1 and #2 and #3 and #4 |

Supplementary Table 3 Characteristics of included literature

| First author | Literature source | Literature theme | Type |
| --- | --- | --- | --- |
| Arends (2017) [17] | Pubmed | ESPEN guidelines on nutrition in cancer patients | Guideline |
| SFNEP (2014) [10] | Pubmed | Clinical nutrition guidelines of the SFNEP | Guideline |
| McClave (2016) [11] | Pubmed | Nutrition therapy in the adult hospitalized patient | Guideline |
| Muscaritoli (2021) [3] | Web of Science | Clinical nutrition in cancer | Guideline |
| CSPEN (2017) [6] | Web of Science | Nutritional support in patients with tumor | Guideline |
| Thompson (2017) [12] | Web of Science | Oncology nutrition practice guideline for adults | Guideline |
| August (2009) [9] | Web of Science | Nutrition support therapy during adult anticancer treatment and in hematopoietic cell transplantation | Guideline |
| Cui (2020) [18] | CNKI | Guidelines for tumor immunonutritional therapy | Guideline |
| CACA (2016) [5] | CNKI | Nutritional therapy guidelines for chemotherapy patients | Guideline |
| CACA (2016) [19] | CNKI | Guidelines for lung cancer nutrition | Guideline |
| CACA (2016) [20] | Wanfang Database | Nutritional treatment guidelines for colorectal cancer | Guideline |
| CACA (2023) [21] | Wanfang Database | The updated guidelines for oral nutritional supplements | Guideline |
| Carrato (2022) [22] | Pubmed | Clinical nutrition as part of the treatment pathway of pancreatic cancer patients | Expert consensus |
| Chen (2018) [23] | Pubmed | Nutrition therapy in esophageal cancer | Expert consensus |
| Lin (2018) [24] | Pubmed | Nutritional intervention for head and neck cancer patients undergoing chemoradiotherapy | Expert consensus |
| CSPEN（2017）[25] | CNKI | Consensus of experts on adult oral nutrition supplements | Expert consensus |
| Zhang (2022) [26] | CNKI | Nutritional management of appetite loss in cancer patients | Expert consensus |
| Li (2016) [27] | CNKI | Application of special medical use formula foods for tumor patients | Expert consensus |
| CSNO (2021) [28] | CNKI | Nutritional therapy for breast cancer patients | Expert consensus |
| CSNO (2021) [29] | Wanfang Database | Nutritional therapy for nasopharyngeal cancer patients | Expert consensus |
| CSNO (2022) [30] | Wanfang Database | Nutrition therapy for pancreatic cancer patients | Expert consensus |
| CSNO (2022) [31] | Wanfang Database | Nutritional therapy for hematologic cancer patients | Expert consensus |
| CSNO (2023) [32] | Wanfang Database | Nutritional therapy for lung cancer patients | Expert consensus |
| CSNO (2023) [33] | CBMdisc | Nutritional therapy for gastric cancer patients | Expert consensus |
| Benna-Doyle (2024) [34] | Pubmed | Nutritional interventions during treatment for ovarian cancer | Systematic review |
| Bossola (2015) [35] | Pubmed | Nutritional interventions in head and neck cancer patients undergoing chemoradiotherapy | Systematic review |
| Bossola (2022) [36] | Pubmed | Tube feeding in patients with head and neck cancer undergoing chemoradiotherapy | Systematic review |
| Caillet (2017) [37] | Pubmed | Association between cachexia, chemotherapy and outcomes in older cancer patients | Systematic review |
| Cintoni (2023) [38] | Web of Science | Nutritional interventions during chemotherapy for pancreatic cancer | Systematic review |
| Dambros (2023) [39] | Web of Science | The effect of oral dietary interventions on nutritional status and treatment tolerance in patients with hematologic neoplasms receiving chemotherapy | Systematic review |
| Heilfort (2023) [40] | Web of Science | Benefit of B-vitamins as a complementary treatment in cancer patients | Systematic review |
| Kikomeko (2023) [41] | Web of Science | Short-term fasting and fasting mimicking diets combined with chemotherapy | Systematic review |
| Limon-Miro (2017) [42] | Web of Science | A critical review of dietary guidelines for breast cancer patients | Systematic review |
| Mello (2021) [43] | Web of Science | Effect of oral nutritional supplements with or without nutritional counseling on head-and-neck cancer patients undergoing (chemo)radiotherapy | Systematic review |
| Miller (2022) [44] | Web of Science | Impact of enteral immunonutrition on infectious complications and immune and inflammatory markers in cancer patients undergoing chemotherapy | Systematic review |
| Newell (2021) [45] | Web of Science | N-3 long-chain polyunsaturated fatty acids, eicosapentaenoic and docosahexaenoic acid, and the role of supplementation during cancer treatment | Systematic review |
| Sadeghian (2021) [47] | Embase | Fasting effects on the response of cancer to chemotherapy | Systematic review |
| Tan (2022) [48] | Embase | Effects of immunonutrition in head and neck cancer patients undergoing cancer treatment | Systematic review |
| Zeidler (2024) [50] | Embase | Impact of nutrition counseling on nutrition status in patients with head and neck cancer | Systematic review |
| Tao (2022) [49] | Embase | Efficacy of ω-3 polyunsaturated fatty acids in patients with lung cancer undergoing radiotherapy and chemotherapy | Meta-analysis |
| Wang (2023) [52] | Cochrane Library | Effects of omega-3 PUFA-enriched oral nutritional intervention on cancer patients receiving chemotherapy | Meta-analysis |
| Zhang (2021) [53] | Cochrane Library | Prognostic nutritional index as a prognostic factor in lung cancer patients receiving chemotherapy | Meta-analysis |
| Zheng (2020) [54] | Cochrane Library | Effects of immunonutrition on chemoradiotherapy patients | Meta-analysis |
| de van der Schueren (2018) [46] | Web of Science | Oral nutritional intervention on nutritional and clinical outcomes during chemo(radio)therapy | Meta-analysis |
| Sa-Nguansai (2024) [51] | Embase | Efficacy of oral nutritional supplement in cancer patients receiving chemotherapy | Meta-analysis |
| Chen (2023) [55] | CNKI | The efficacy of Omega-3 PUFA in chemotherapy patients | Meta-analysis |
| Wei (2021) [56] | CNKI | The efficacy of immunonutrition in gastrointestinal cancer patients undergoing chemotherapy | Meta-analysis |

Abbreviations: ESPEN, European Society for Parenteral and Enteral Nutrition; SFNEP, French Speaking Society of Clinical Nutrition and Metabolism; CNKI, China National Knowledge Infrastructure; CBMdisc, China Biology Medicine disc; CSPEN, Chinese Society for Parenteral and Enteral Nutrition; CACA, China Anti-Cancer Association; CSNO, Chinese Society of Nutritional Oncology; PUFA, polyunsaturated fatty acids.
